# Encountering patients in grief due to the death of a loved one: nurses’ experiences in municipal home care – a qualitative study

**DOI:** 10.1177/17449871261442439

**Published:** 2026-05-15

**Authors:** Anne-Lie Larsson, Eva-Lena Einberg, Ann-Christin Janlöv, Ingela Beck

**Affiliations:** The Research Platform for Collaboration for Health, Faculty of Health Sciences, Kristianstad University, Kristianstad, Sweden; Senior Lecturer, The Research Platform for Collaboration for Health, Faculty of Health Sciences, Kristianstad University, Kristianstad, Sweden; Associate Professor, The Research Platform for Collaboration for Health, Faculty of Health Sciences, Kristianstad University, Kristianstad, Sweden; Associate Professor, The Research Platform for Collaboration for Health, Faculty of Health Sciences, Kristianstad University, Kristianstad, Sweden; Institute for Palliative Care, Lund University and Region Skåne, Lund, Sweden

**Keywords:** grief, home care service, homes for the aged, nurses, patients, qualitative research

## Abstract

**Background:**

Registered nurses (RNs) in municipal home care (MHC) encounter patients with illnesses and/or disabilities. When these patients suffer a stressful event, such as the loss of a loved one, there is an increased risk of suffering further ill health. It is unclear whether RNs are aware of patients’ grief and how to support and promote their well-being and health.

**Aims:**

The aim was to explore RNs’ experiences of paying attention to and encountering patients receiving MHC, who are grieving due to the death of a loved one.

**Methods:**

This is an exploratory qualitative study with focus groups (*n* = 2) and individual interviews (*n* = 13) with RNs (*n* = 22) in MHC. The data were analysed inductively through reflexive thematic analysis.

**Results:**

The results show three themes: *Becoming aware that the patient’s health is declining, Feeling responsibility for listening to memories and experiences of loss* and *Dealing with one’s own emotions*, with six corresponding sub-themes.

**Conclusion:**

RNs encountered emotional patients in grief, while they themselves felt inadequate and emotionally affected. Further training in grief and communication, along with organisational support that enables sufficient time with patients and interprofessional collaboration and reflection, is needed to enable holistic, person-centred care.

## Introduction

Registered nurses (RNs) constitute one of the largest professional groups within the global healthcare workforce and play a central role in promoting health, preventing disease and alleviating suffering throughout the life course (World Health Organization [WHO], 2025). With an estimated 63 million deaths worldwide in 2025 (Our World in Data, 2025), grief emerges as a common experience across cultures and healthcare contexts, highlighting the frequent encounters with death and grief in nursing practice. Grief is commonly understood as affecting relatives following a patient’s death. However, patients themselves may also experience grief after losing a close loved one, a form of grief that risks being overlooked despite its significance for patients’ healthcare and nursing needs.

In Sweden, municipalities are responsible for delivering home healthcare services to individuals living in both ordinary housing and residential care facilities who are unable to independently access a primary healthcare centre (The National Board of Health and Welfare, 2019). Within municipal home care (MHC), RNs encounter patients in grief in different care contexts. Regardless of whether RNs work in residential care facilities or visit patients in their own homes, they care for patients with illness or disability. A large proportion of these patients are older persons, with comorbidities and particularly fragile (Gustafsson et al., 2022). People living with illness and/or disability have a diminished ability to handle highly stressful situations (WHO, 2017), such as bereavement through the death of a loved one.

Although grief due to bereavement is considered a natural experience during a lifetime (Parkes, 1998; Stroebe et al., 2007a), it poses considerable health risks since it involves psychobiological stress reactions that affect both mental and physical health negatively. Grief means an increased risk of developing depression (Maercker and Lalor, 2022), anxiety, post-traumatic stress disorder or cognitive difficulties (Mason et al., 2020), as well as suffering from cardiovascular diseases and disorders of the immune system (Buckley et al., 2022). In the worst case, it can lead to death, such as broken heart syndrome (Fagundes and Wu, 2020) or suicide (Zisook and Shear, 2009). People who already have illnesses and disabilities are at significant risk of health deterioration when they are faced with overwhelming life events, such as grief. It is unclear to what extent RNs working in MHC are aware of patients’ grief and its psychobiological consequences, which are a prerequisite for providing the appropriate nursing that these patients need.

A study by Van Humbeeck et al. (2016a) found that persons living in residential care facilities described feeling invisible and discouraged by those around them from being present at their loved one’s deathbeds. When death occurred, they felt unprepared and alone in their grief. Sjöberg et al. (2018) showed that persons receiving long-term care experienced a lack of someone with whom to share their thoughts and sorrow, which evoked a deep sense of loneliness. Involuntary social isolation and loneliness are unhealthy (Leigh-Hunt et al., 2017; Park et al., 2020) and being alone and isolated can evoke feelings of sadness and emptiness, and life can feel meaningless (Gil Álvarez et al., 2023).

Individuals experiencing grief require recognition and acknowledgement of their grief. A report drawing on bereavement counsellors’ experiences shows that people experiencing grief need someone to talk to and be listened to, for a sense of meaning (Carlsson, et al., 2022), and need space to talk about the deceased (Aoun et al., 2019). In this context, RNs have a central role and responsibility to meet patients’ needs for nursing care and to provide supportive interventions (International Council of Nurses [ICN], 2021), including in situations of grief. Despite these important aspects, studies specifically exploring RNs’ experiences of caring for patients in grief are limited. However, a qualitative study from Belgium, in which the results do not distinguish between RNs and nurse assistants (NAs), showed that coping with grief can be challenging, for example, in recognising grief and how to provide professional care (Van Humbeeck et al., 2016b).

RNs in Sweden and many European countries have a 3-year university education (Taneva et al., 2023). The professional responsibility of the RN is to provide nursing care and promote the health of patients in primary healthcare based on a person-centred approach (ICN, 2021). Such responsibility implies that RNs are present in the care encounter and adopt a holistic approach, which includes a salutogenic and pathological perspective. Such care encounters require not only knowledge but also emotional and physical presence to provide professional support (Haugan and Eriksson, 2021). However, a study by Sundström et al. (2018) showed that RNs feel insecure about encountering patients’ existential issues, including thoughts about life and death. To sum up, bereaved patients are at significant risk of health deterioration, which highlights their crucial need for therapeutic nursing support. The RN is responsible for providing nursing care and promoting patients’ health based on a person-centred approach. Additionally, they are responsible for supporting team members, such as NAs, in providing support to patients experiencing grief. The extent to which RNs are aware of and attentive to patients’ grief in MHC is an area that has not been thoroughly examined. Therefore, this study aimed to explore RNs’ experiences of paying attention to and encountering patients receiving MHC, who are in grief due to the death of a loved one.

## Methodology

### Design

The study took an exploratory qualitative design with data triangulation, collecting data sequentially through focus groups (FGs) followed by individual interviews. Reflexive thematic analysis with an inductive approach was used to analyse the data, as this method acknowledges the researcher’s active role and enables an in-depth understanding of participants’ experiences and meanings (Braun and Clarke, 2019). A reference group was included to incorporate a user perspective (Bammer, 2019) and was involved in pilot-testing the interview guide and providing feedback on the preliminary results. The reference group consisted of an RN, an NA (a care assistant with upper secondary education who provides social service and daily care, and who performs delegated healthcare tasks under the supervision of the RN), a deacon (a professional working in the religious community providing pastoral and psychosocial support) and a person who had experienced grief after the death of a loved one. One of them was a man. This study followed the guidelines of Braun and Clarke (2024) and is reported in accordance with the Consolidated Criteria for Reporting Qualitative Research (COREQ) guidelines (Tong et al., 2007), where applicable.

### Context and setting

This study was conducted in six municipalities in southern Sweden, with populations ranging from 7,900 to 86,600 inhabitants. MHC is mainly financed through income taxes and government grants (Government Offices of Sweden, 2021). The RNs have overall responsibility and a leading role in nursing care. They manage many fragile patients with multiple diagnoses, and their work is described as consultative (Norell et al., 2013), which means without daily contact with patients. The RNs collaborate with professionals employed by the municipality – including NAs, occupational therapists, physiotherapists and needs assessors – and with physicians employed in the region’s primary care. Outside the region and municipal organisation, counsellors and religious representatives can be contacted, if needed. The RNs work in various care units, including residential care facilities (RCFs), where they provide long-term care for patients. They also work in short-term care (STC) for patients before transitioning to a residential care facility or returning home. These facilities are staffed by NAs 24 hours a day. In addition, RNs work in home care (HC), providing care in patients’ homes with HC teams (HCTs), giving ongoing care for a limited duration, based on hospitals’ individualised discharge plans. The RNs are usually on site on weekdays and on-call at other times. Therefore, they need to delegate responsibility to the NAs, such as medication administration (Craftman et al., 2013).

### Participants

The study used purposive sampling with maximum variation to select participants (*n* = 22) from different care contexts in six municipalities to provide a variety of experiences (Flanagan and Beck, 2024). Inclusion criteria required RNs to have experience of patients in grief and to be employed in MHC. The educational backgrounds included undergraduate or postgraduate degrees, with different specialisations (Table 1). The heads of each care unit assisted in disseminating written information and a video presentation about the study. Furthermore, at workplace meetings, the first author informed RNs about the study and invited participation. Participants indicated interest by completing a response letter during the workplace meeting, emailing the first author, or contacting workplace heads. Then each RN called to confirm participation or ask questions. In total, 23 RNs initially expressed interest in participating. Two later withdrew due to a lack of experience with patients in grief. One participant joined an FG through contact with another participant without having previously expressed interest and participated after providing informed consent. This resulted in a final sample of 22 participants. The researchers had no prior relationship with the participants before the study began.

**Table 1.** Characteristics of participants.

Characteristics	*n* = 22
Gender, women/men	21/1
Age, mean (range)	47 (26–65)
Native language other than Swedish Specialist nurse education in: District healthcare/elderly care/psychiatry	3 5/2/1
Education in grief, yes/no	4/18
Workplace, HC/HCT/RCF/STC	11/1/9/1
Municipality, A/B/C/D/E/F	3/6/2/2/3/6

HC: home care; HCT: home care team; RCF: residential care facilities; STC: short-term care.

### Data collection

Two FGs and 13 individual interviews were conducted between November 2022 and September 2023. The same semi-structured interview guide with open-ended questions was used in the FGs and individual interviews, with minor adjustments to suit the interview formats (Available as supplementary material). The opening question was: Would you like to share your experience of encountering a patient in grief when their loved one had died? The interview guide was pilot-tested with the reference group to ensure the questions were understandable and relevant and were adjusted appropriately. This interview is not part of the study. Firstly, the FGs were conducted with participants by the first and second authors in two of the municipalities (A [*n* = 3], B [*n* = 6]), to discuss and gain an understanding of their experiences (Krueger and Casey, 2015). The first author acted as a moderator, whereas the second author took the observer role, asking clarifying questions and ensuring that all participants were able to contribute to the discussion. The FGs lasted just over 60 minutes. Afterwards the FGs, individual interviews were conducted with participants from other municipalities to gain a deeper and detailed account of experiences (Lambert and Loiselle, 2008). The individual interviews were carried out by the first author and lasted 30–60 minutes. The FGs and individual interviews were conducted at the RNs’ workplace in a separate, quiet room and were audio-recorded and transcribed.

### Data analysis

The transcribed data were inductively analysed using reflexive thematic analysis according to Braun and Clarke (2019). This approach involved the researcher engaging in self-reflection during the analysis process, investigating how assumptions could affect the interpretation of the data. This was achieved by documenting reflexive thoughts through written analytic memos and ongoing author discussions informed by diverse specialist backgrounds in nursing. For example, an early analytic assumption was that RNs did not reflect on grief; further engagement with data revealed that such reflections did occur in collaboration with colleagues. The non-linear analysis followed six phases (Braun and Clarke, 2006) see Tables 2 and 3. Theoretical interpretation was applied during the writing of the discussion.

**Table 2.** Overview of the analysis process.

Phase 1, Familiarisation	Phase 2, Generating initial codes	Phase 3, Searching for sub-themes	Phase 4, Reviewing themes	Phase 5, Defining and naming themes	Phase 6, Producing the report
The first author read the transcripts several times, noting initial patterns and ideas. These were reflected upon and discussed among all authors	The first author reread the transcripts and marked relevant text with initial codes. Coding and extracted data were discussed with the last author	The first author sorted codes with similar content and began developing potential sub-themes. Sub-themes were discussed and grouped into themes with all authors	Temporary sub-themes and themes were reviewed and compared with the coded extracts and the entire data set for consistency	Sub-themes and themes were refined, named and finalised through discussions among all authors	The first author wrote the analytical report, which was discussed with all authors. Preliminary results were presented to a reference group for validation before finalising the analysis

**Table 3.** Examples from the analysis process.

Data extracts	Initial codes	Sub-themes
(RN A) Yes, like when you [the patient] is. . . you see the hopelessness and all that. (RN B) Yes. . . (RN C) Yes, he sat by her [his late wife] bed and. . . he was quietly sad, if you know what I mean, like it was not. . . anything hysterical or. . . but still, it was calmly. (FG 1)	Sees the patient’s hopelessness. Sitting by his late wife’s bed, he was quietly sad — not hysterical, but calm in a way	Facing distressing feelings and thoughts of life and death
Yes, she [the patient] did. . . she became worse in her illness [dementia when her husband passed away], I would say. /. . ./ In the end, she more or less gave up. (Interview 11)	The patient’s illness worsened [after her husband’s death], she more or less gave up	Facing mental and bodily deterioration
So I sat down and, well, asked, how are you, how is it going, I mean, asked a few questions [to the patient] and then she. . . it is not like it comes right away /. . ./ but rather. . . little by little. (Interview 10)	The nurse sat down, asked the patient how she was and how things were going. [The patient] does not respond directly, but [talks] little by little	Showing concern and space for dialogue
And he [the patient] talked a lot about his wife [deceased] when I was there /. . ./ Yes, and he started crying now and then, several times, and said, oh, I miss her so much. (Interview 4)	The patient talked a lot about his wife, cried several times at intervals and said that he misses her	Becoming increasingly aware of the need to talk about the loss
He was upstairs; they lived in a house /. . ./ But then I said to the wife that at least I must go up and see [if he is dead]. And sure enough, that was the case. But then I went back downstairs, and she poured coffee and. . . for her, it was completely natural, he had. . . he was dead, as she said. (Interview 6)	I said to the wife [patient] that I at least must go up to see if he is dead. The wife had poured the coffee; it seemed completely natural	Become emotionally touched
No, it is not easy to find . . . to know what you can talk about, because you do not want to bring up too many things that reminded of the late wife. (Interview 11)	It is not easy to know what to talk about, not wanting to bring up things that remind him of his late wife	Feeling inadequate in dealing with others’ grief and needing training

RN: registered nurse.

### Trustworthiness

The trustworthiness of the study was addressed in accordance with Lincoln and Guba’s (1985) criteria of credibility, dependability, confirmability and transferability. Credibility was strengthened using both FGs and individual interviews. Dependability was supported by a transparent description of the analysis process, including its six phases. Confirmability was enhanced using written analytic memos and continuous reflective discussions among the authors throughout the research process. Transferability was supported by providing a detailed description of the study context and the participants.

### Ethical consideration

The study received ethical approval from the Swedish Ethical Review Authority (Dnr. 2021-03705) and followed the procedures according to the Declaration of Helsinki (World Medical Association, 2024). Participants received verbal and written information about the study and the voluntary aspect of participation. They were also informed that they could withdraw without a reason and that the interview material would be treated confidentially. The transcribed interviews were coded and securely stored.

## Results

Overall, the RNs in MHC, regardless of workplace, had largely similar experiences of paying attention to and encountering patients in grief due to the death of a loved one. During planned nursing or medical interventions, such as home dialysis, dressing wounds or taking blood samples, they were also attentive to patients’ mental state and behaviour. They described patients in mourning for deceased loved ones who were often spouses, life partners or children whose death occurred after prolonged long-term illness, sudden illness, accident or suicide. The results are generated in three themes with associated sub-themes (Table 4).

**Table 4.** Overview of themes and sub-themes showing RNs’ experiences of paying attention to and encountering patients who are in grief.

Themes	Sub-themes
Becoming aware that the patient’s health is declining	Facing distressing feelings and thoughts of life and death Facing mental and bodily deterioration
Feeling a responsibility for listening to memories and experiences of loss	Showing concern and space for dialogue Becoming increasingly aware of the need to talk about the loss
Dealing with one’s own emotions	Being emotionally touched Feeling inadequate in dealing with others’ grief and needing training

RN: registered nurse.

## Becoming aware that the patient’s health is declining

*Facing distressing feelings and thoughts of life and death* awakened an awareness that the patients were grieving. The RNs encountered patients expressing anger and frustration. They could also express their grief by crying wildly, becoming breathless as they confronted the reality of their loved one’s death.

So, she [the patient’s husband passed away] got sad right away, she did. /. . ./ And kind of a bit of that panic feeling. /. . ./ Yes, it was more about the way she cried, or how to put it, you know that thing when. . . you just can’t quite catch your breath. (Interview 10)

The RNs noticed that the expressions of grief among patients varied, showing the different circumstances of each death. Losses could be unexpected, sudden or unexplained. Painful distress might arise immediately after the loss but might also resurface even several years later. The RNs described that some patients tried to hold back their sorrow, but the suffering was present in their facial expressions and eyes.

Furthermore, the RNs described situations in which they heard patients verbally express an inner turmoil and a feeling of pressure in their chest. They also expressed sorrow and depression because the loved one was no longer alive. The RNs heard patients express feelings of loneliness and thoughts about death and living at home alone. A deep feeling of loneliness was described as a sense of emptiness. The RNs also heard patients say that they had not received visitors for a long time or had been unable to leave their homes. The burdening emptiness appeared, according to the RNs, regardless of whether family and friends were nearby. Patients expressed their loss as though a part of themselves was gone when a life partner passed away. The RNs also described that they heard patients talk about their own death. For example, patients would lie in bed, looking at the empty bed where their deceased loved one had slept and express a sense that their death was imminent. RNs also sometimes heard patients express a desire to no longer live, as they felt life had lost its meaning. The RNs realised the impact grief had on the patients’ health, causing them to fear that the patients were planning suicide.

. . .Then he mentioned that he didn’t want to live anymore, and he had weapons at home in his gun cabinet, because he had been a hunter, so that became another part of it, that there was a risk of suicide. (Interview 8)

The RNs heard that patients could question why younger loved ones, such as children, had to die from illness or suicide. These patients expressed feelings of being finished with life and said that it should have been their turn to die, instead of their younger loved ones. Patients discharged from STC told the RNs that they no longer wanted to live at home after losing their life partner. Conversely, some were forced to leave their homes due to a decline in support after the death of a spouse, both in complementary care and economic resources, forcing patients to move to RCFs.

*Facing mental and bodily deterioration* made RNs aware of the patient’s declining health. The RNs encountered patients who repeatedly questioned whether their loved one had truly passed away and how the death had occurred, despite being informed previously. These questions could arise every time the RNs visited the patients. Patients experienced difficulty absorbing new information or instructions, such as explanations about their medical condition or treatment plan.

The RNs also described instances where, during visits, patients remained in bed and appeared to speak to someone not physically present in the room, interpreted as conversations with their deceased loved ones. Patients might stay in bed or sit in an armchair with a blank stare, even when RNs tried to get attention by talking to patients or touching them. In such cases, the patient looked away, seemingly retreating into a shielded state. In addition, the RNs observed that after the loss, patients withdrew from social interactions, becoming more isolated and sedentary at home. Following their loss, patients could develop depression, sometimes needing medical treatment. In cases where medical treatment was ineffective, the patient’s condition could deteriorate so that they needed end-of-life care, and they eventually died. In such cases, the RNs encountered the patients’ grief and ultimately, their death.

They also observed how patients’ bodies deteriorated in various ways, due to poor appetite and dehydration, which resulted in dizziness and falls. RNs described that it could even be difficult to get the deteriorating patients to accept they were the same person as before the loss. The deterioration could happen fast. It often exacerbated the patients’ underlying medical conditions, leading to further decline.

From the state you were in . . . You gave up /. . ./ Yes, the person, I am thinking of sounded. . . and since giving up, the illness took a strong hold, and it didn’t take long before she passed away. (Interview 12)

## Feeling responsibility for listening to memories and experiences of loss

*Showing concern and space for dialogue* was a way to respond to and alleviate patients’ suffering. According to the RNs’ experiences, it was important to take time to sit with the bereaved, especially when a spouse had recently died. In this situation, the RNs tried to show empathy by being close, making eye contact and giving reassurance.

His grief isn’t something we can solve, other than by listening to him, but when he starts to drift away a little, you try to find his eyes again, maybe place a hand on his arm, and say, Yes, but we’ll get through this. (Interview 14)

Furthermore, the RNs tried to create space for dialogue, to ease the patients’ pain and sorrow by asking questions about how they were feeling and if they needed support. The RNs found it easier to start conversations about emotional topics during care interventions that required more time, such as time-consuming wound dressings and home dialysis. These moments were an opportunity to build trust and a relationship, leading to both meaningful and longer conversations. The conversation with patients could be initiated during home visits, such as when providing information about medical treatment in person rather than by phone, in the hope of easing their grief. Sometimes, RNs found it difficult to sit down and talk with patients due to time constraints. Instead, patients were encouraged to seek contact with RNs themselves and request support when needed. For the RNs, it was important to be available and present for the bereaved when they required support.

*Becoming increasingly aware of the need to talk about the loss*, concerned when the patients began to talk about their life with the deceased loved ones and speak about the loss. The RNs listened to patients’ memories about their spouses or life partners, such as decorating their shared home together, and remembering the happy times they had experienced over the years. The moments also included memories of family trips taken over the years or relocating together to another city for work-related reasons. When listening to these stories, the RNs became aware of the bereaved patients’ need to share their experience with someone, and it seemed to help ease their painful sorrow.

The RNs found that patients often wanted to talk about their deceased spouse or partner and their loss, a topic that came up repeatedly during home visits. During these talks, it was not unusual to be shown photos of their loved one, and the RNs could see the sorrow of bereavement in the patient’s face.

No, but he talks about how they [the patient and his wife] have had it in life and what they have done and so on, because it really seems like talking about it somehow makes him feel calmer. (Interview 15)

## Dealing with one’s own emotions

*Being emotionally touched* concerns meeting patients who were suffering from grief due to losing a loved one. Being emotionally affected reflects RNs’ experiences of caring for patients who were grieving and how these encounters also affected them emotionally. The RNs found that they were drawn into the grief, sometimes feeling down themselves, when they saw that the patient was noticeably affected by the spouse’s death. The RNs could miss the pleasant moments spent with patients and their loved ones before the death, but after the death, all that disappeared. These feelings that arose stayed with the RNs after the visit was over. The RNs noted that they were touched when patients shared with them how they lost their loved one.

(RN A) She had found her son’s suicide note in one of the moving boxes, then she showed it to me and said, Look at what he wrote to me. . ./. . ./. It was incredibly sad in every way, but I think. . . it was maybe forty years ago, so she, in some way, could handle it, but I think I, as an RN, was more affected. (RN B) /. . ./I meet many patients, and it often comes up that they have lost a child, or an adult child, or someone else over the years. . . (FG 2)

Conversely, the RNs described that they found it confusing when they encountered patients reacting unexpectedly after the loss, for example, in a situation where the reaction was that they were not allowed to refer to the deceased by name. At the same time, the patient seemed emotionally unaffected by the fact that their life partner had passed away. This lack of visible grief felt strange and was challenging for the RNs to process.

*Feeling inadequate in dealing with others’ grief and needing training* arose when encountering bereaved patients. The RNs expressed feeling pressured to talk to grieving patients in a constructive way, both by themselves and others. They also felt unable to talk to the bereaved because they did not know what was appropriate to say. The RNs described struggling with how to address emotional topics, such as losing a spouse, without making the situation worse.

The feeling of inadequacy was evoked when RNs realised their ability to provide conversational support to alleviate patients’ grief was limited. In these situations, they could contact a priest, deacon, counsellor or physician, especially when they noticed that patients seemed stuck in grief and needed additional conversational support or medical treatment. Furthermore, the RNs described instances when their support felt insufficient and they witnessed patients’ sorrow weighing them down so much that they could not cope with everyday life. In such cases, the needs assessor was contacted to plan additional assistance from NAs. The RNs noted that the NAs usually had closer relationships with patients due to their daily involvement in care and familiarity with the patients, making it easier for them to communicate and understand the bereaved person’s needs.

I find it quite difficult [to handle grief] as a nurse, because most of the time, they have a completely different relationship, nurse assistants, with the patient, which means they are better at reading her [the patient] and how she is doing. (Interview 10)

When the RNs felt unable and inadequate to encounter and cope with the patients suffering from grief, support from colleagues became crucial. Sharing experiences and reflecting together on how to address grief made it easier for them to encounter bereaved patients. The RNs also expressed a need for further training in the bereavement process and how to care for mourning patients to help reduce their pain. Furthermore, they expressed a desire to be able to develop their ability to talk with bereaved patients about sensitive topics related to their grief with the bereaved patients.

The RNs also emphasised the need to gain knowledge from professionals within religious organisations who meet persons in grief, such as priests or deacons, to better recognise how to support patients suffering painful feelings, from different backgrounds, experiencing the grieving process.

## Discussion

Our results showed RNs’ awareness of patients’ grief due to loss of loved ones. The RNs noticed patients’ grief by being present and listening to their descriptions of distressing feelings and thoughts and became aware of the patients’ suffering and their health decline. They felt a responsibility and strived to alleviate their suffering, but felt inadequate in dealing with patients’ grief. The findings can be understood in the light of the work of Levinas (1979), who argued that coming face to face with a vulnerable person’s suffering evokes an ethical obligation. When the RNs witnessed the patients’ suffering, this ethical responsibility became evident. At the same time, they felt inadequate and in need of increased competence in dealing with the patients’ grief, which relates to the prerequisites for and practical conditions surrounding care. Therefore, the findings can also be interpreted through McCance and McCormack’s (2021) framework for person-centred practice, which emphasises that certain conditions are required for RNs to be able to provide person-centred care that is healthy for patients, but also for the RNs. Person-centred care for patients in grief entails person-centred processes such as working with their beliefs and values, engaging authentically, sharing decision-making, being sympathetically present and providing holistic care. According to the framework, such care requires not only RNs with professional competence and developed interpersonal skills but also a supportive organisational system.

RNs’ ethical responsibility is evoked when being sympathetically present in the care meetings with patients suffering due to grief. Our results showed that RNs used their senses to perceive and respond to expressions of distress, showed concern and tried to give patients space for a dialogue. Being attentive to patients’ expressions of distress could be seen as an ethical obligation (Levinas, 1979), one that emerges in the encounter with the patient and constitutes a central aspect of professional nursing care, grounded in a relational, ethically informed person-centred nursing (McCance and McCormack, 2025). Such attentiveness is of importance in the care meeting and can be linked to sympathetic presence, one of the person-centred processes (McCance and McCormack, 2021). Such presence is a way of being that recognises the uniqueness and values of the other person. It entails being adaptively attentive and emotionally open to the other person’s feelings, experiences, characteristics and suffering in the moment (McCance et al., 2021). Similarly, Larsson Gerdin et al. (2021) found that encounters with patients in MHC involved being receptive to the other. Recognising expressions of values and experiences is important for customising person-centred care but is not enough to ensure that health is promoted (McCance and McCormack, 2021).

During home visits, the RNs became aware of the negative consequences of grief on the patients’ health and endeavoured to provide support and alleviate suffering. The results show that RNs observed both strong grief reactions and more silent forms of sorrow, and some patients expressed existential thoughts or a desire not to live. This reflects the emotional distress that bereavement evokes and its associated risk of suicide (Stroebe et al., 2007b). The RNs also observed patients with depressed mood, memory difficulties and a general decline in health, and in some cases, noted that patients died shortly after the death of a loved one. These results are in line with research showing that grief increases the risk of depression (Maercker and Lalor, 2022), memory problems (Näsman et al., 2020), permanent cognitive impairment (Pérez et al., 2018), cardiovascular diseases, disorders of the immune system (Buckley et al., 2022) and even death through ‘broken heart’ syndrome (Fagundes and Wu, 2020). Interpreted through McCance and McCormack et al. (2021), the results emphasise the importance of professional competence and preparedness in recognising the health-related consequences of grief. This competence is essential to respond to and provide nursing care based on patient’s emotional as well as physical needs. This links to the practice environment dimension in the framework, which must provide conditions that support RNs in recognising and responding to patients’ needs. Our results show that when RNs observed patients’ ill health, they tried to alleviate suffering by showing closeness and being present. In addition to such immediate support, RNs acted on patients’ grief by initiating conversations that allowed expressions of loss and occasionally involved other professionals. Ribera-Asensi et al. (2025) described the relationship between emotional support during grief and a reduced risk of prolonged grief and ill health. Haugan and Eriksson (2021) further emphasised that the nurse–patient interaction itself is a vital health-promoting resource, highlighting the importance of a holistic approach that addresses pathogenic risks while strengthening salutogenic resources.

Facing patients in grief challenges RNs both emotionally and professionally. Our results show that the RNs often felt insecure about dealing with patients’ grief and experienced pressure both from themselves and others to manage these situations. Uncertainty about addressing emotional and existential questions has also been found in other studies. Sundström et al. (2018) reported that healthcare professionals feel uncertain about how to encounter patients’ existential issues. Jansen et al. (2024) found that RNs in primary care felt apprehensive about engaging in conversations with relatives in grief. Furthermore, Tomstad et al. (2021) reported that RNs in HC find it challenging to have emotional conversations with patients. Encountering patients in grief requires well-developed interpersonal communication skills and increased self-awareness. A study by Bäckersten et al. (2025) found that patients with life-threatening illness and old age considered nurses as discussion partners regarding existential issues, but this requires nurses who have time, courage and can instil trust. Thus, caring and existential conversations are central to nursing care and require attentiveness in their conduct (Öhlén and Friberg, 2023). However, limited time and RNs’ reduced involvement in daily care may limit opportunities to develop and practice the interpersonal skills required for such conversations. Feeling inadequate when encountering patients in grief may limit RNs ability to respond to patients’ needs for conversation, as well as their ability to manage their own emotions, which in turn may increase the risk of compassion fatigue, that is, emotional exhaustion resulting from repeated exposure to emotionally demanding situations (Gustafsson and Hemberg, 2022). Furthermore, this professionally demanding work is exhausting for the RNs, both professionally and privately. To meet patients’ needs in grief also requires organisational support that facilitates reflection and emotional processing (McCance and McCormack, 2021) not only to promote patients’ health but also to avoid jeopardising the RNs’ own health.

From a holistic perspective, Dossey (2008) emphasised that death is a natural part of life, meaning that death and related aspects are part of what nursing must deal with. It requires RNs to develop their inner, private self through a reflective practice, approaching aspects of death, dying and even imagining one’s own death. However, our results show that RNs involve NAs in supporting patients’ grief-related needs, even though NAs have less formal training in this area because NAs provide daily care and therefore have closer relationships with patients than RNs do. Larsson et al. (2024) found that NAs working in municipal health and social care experience difficulties in approaching and meeting the needs of patients in grief. This suggests that neither RNs nor NAs feel equipped to respond to the needs of patients in bereavement, placing these patients at risk of not receiving the support they need.

From a holistic nursing perspective, supporting patients in grief requires time for relational engagement. Person-centred nursing care requires time spent with patients to understand what matters to them (McCance and McCormack, 2025). However, organisational structures often distance RNs from direct patient care, thereby limiting the relational aspects of the RN’s professional role. Shariff et al. (2017), in a narrative literature review, emphasised the importance of providing RNs with time and space to reflect on and develop awareness of their own perceptions of death and dying to optimally support persons in grief, support NAs and maintain their own professional well-being. Although reflection is essential for RNs to understand their own perceptions of death and dying (Shariff et al., 2017), repeated communication training and reflections grounded in person-centred values can further enhance their confidence and ability to engage in meaningful conversations with patients in grief. A study by Laustsen et al. (2025) highlights that communication training integrated into RN and specialist RN education strengthened students’ awareness, self-confidence and ability to communicate with patients in need of care. Creating organisational, educational and individual opportunities for continuous professional development can enhance RNs’ capacity to provide meaningful support and promote patients’ holistic well-being. This underscores the need for interaction between individual competencies and supportive organisational conditions to achieve truly person-centred care (McCance and McCormack, 2021).

### Implications

Taken together, these findings point to implications for education, clinical practice, healthcare systems and future research. Nursing education should incorporate structured training in grief support to be able meet biopsychosocial needs, and person-centred communication, whereas specialist nurse programmes should also integrate practical training in person-centred nursing leadership. In clinical practice, training in assessing care needs and addressing grief, together with structured reflective supervision for healthcare teams, should be implemented. At an organisational level, nurses’ working conditions need to be changed to ensure that they are given sufficient time with patients to assess and understand their needs and to provide holistic care based on identified needs. There is a need for further research exploring patients’ needs, wishes and preferences regarding grief support interventions in MHC.

### Strengths and limitations

The strengths of this study were triangulation (Lincoln and Guba, 1985), that is data and site. Sequential data collection, with FG followed by individual interviews, enabled the exploration of specific experiences in greater depth and the narrations of personal experiences (Morgan, 1996). The interviewers strove to be attentive and create a permissive atmosphere during the interviews to strengthen the study’s trustworthiness (Lincoln and Guba, 1985). However, the participants expressed that the interviews provided a valuable opportunity to reflect on work-related experiences of grief, allowing them to articulate and elaborate on their experiences, thereby contributing to the depth and richness of data. Another strength was the use of a reference group who were helpful in pilot-testing the interview guide and giving feedback on the preliminary results. There are also limitations, such as the under-representation of men among the participants in the study, and all authors were women, which may limit the transferability of the findings to men. This reflects the well-known over-representation of women in the RN profession, which was expected. Variation among participants from six geographically diverse municipalities, together with the thick description, contributes to enabling readers to assess the transferability of the findings to similar settings (Lincoln and Guba, 1985).

## Conclusion

RNs encountered emotionally affected patients in grief who expressed loneliness and meaninglessness in their lives after losing a loved one. RNs also observed declining health and sought to relieve suffering through presence and conversation. At the same time, RNs felt inadequate, struggled to talk about loss and were themselves emotionally affected. To ensure that patients’ needs are met and to avoid compromising RNs’ health, RNs require education in grief processes, including normal and complicated grief, and training in person-centred communication. Organisational structures are needed to support sufficient time with patients and interprofessional teamwork and reflection to enable a holistic assessment and addressing of patients’ physical, psychological, social and existential needs.

**Key points for policy, practice and research**

- RNs noted that grief had an impact on patients’ health, both mentally and physically, leading to poorer health and increased risk of mortality.
- RNs attempted to create space for conversations about loss and grief to alleviate patients’ suffering.
- Encountering patients in painful grief affected RNs emotionally and led to a sense of inadequacy. The nurses experienced difficulties in talking about grief and expressed uncertainty about how they could best support the patients.
- RNs requested additional training in conversational methodology and about what grief means to better manage patients in grief.
- At both individual and organisational levels, RNs require sufficient time for relational care, access to education on grief and its biopsychosocial consequences and leadership practices that acknowledge and support the emotional labour involved in caring for patients in grief.

## Supplemental Material

sj-docx-1-jrn-10.1177_17449871261442439 – Supplemental material for Encountering patients in grief due to the death of a loved one: nurses’ experiences in municipal home care – a qualitative study Supplemental material, sj-docx-1-jrn-10.1177_17449871261442439 for Encountering patients in grief due to the death of a loved one: nurses’ experiences in municipal home care – a qualitative study by Anne-Lie Larsson, Eva-Lena Einberg, Ann-Christin Janlöv and Ingela Beck in Journal of Research in Nursing

sj-pdf-2-jrn-10.1177_17449871261442439 – Supplemental material for Encountering patients in grief due to the death of a loved one: nurses’ experiences in municipal home care – a qualitative study Supplemental material, sj-pdf-2-jrn-10.1177_17449871261442439 for Encountering patients in grief due to the death of a loved one: nurses’ experiences in municipal home care – a qualitative study by Anne-Lie Larsson, Eva-Lena Einberg, Ann-Christin Janlöv and Ingela Beck in Journal of Research in Nursing
