# Supplementary material for: Encountering patients in grief due to the death of a loved one: nurses’ experiences in municipal home care – a qualitative study
Source: J Res Nurs. 2026 May 15:17449871261442439. Online ahead of print. doi: 10.1177/17449871261442439 (PMC13179940; doi:10.1177/17449871261442439)
Supplement: sj-docx-1-jrn-10.1177_17449871261442439 – Supplemental material for Encountering patients in grief due to the death of a loved one: nurses’ experiences in municipal home care – a qualitative study [file sj-docx-1-jrn-10.1177_17449871261442439.docx]

**Appendix I.** **Interview guide**

| **Grief-Would you like to talk about:** |
| --- |
| - **What does grief mean to you?** - Can you describe a specific situation related to grief? - What is normal grief or grieving? (Is it the same for everyone? How long does it last?) - Can you give a concrete example of when you met a patient in grief? (Consider time aspects: Before, immediately after, or sometime after the death; whether the death was sudden or expected). |
| **Encountering grief-Would you like to talk about:** |
| - **Your experiences of encountering people in grief after the death of a loved one?** - How do you recognise that a person is grieving (Signs or expressions)? Can you describe a concrete example? - How do you respond to a person in grief? - What do you do when you meet or support a person in grief? - Are there any difficulties or barriers when responding to a person in grief? - What facilitates or helps you when meeting a person in grief? |
| **Needs-Would you talk about:** |
| - **Your experiences of the needs of people in grief?** (Consider time aspects: before, immediately after, or some time following the death; sudden or expected death; changes in life situation). - What according to your experience makes grieving or the grieving process more difficult? (For example, consequences of the death). - What, according to your experience, supports recovery after loss? (Relationships, consequences, or other factors). - How do you identify a person who needs additional support in their grief? - What are your experiences of other professionals or care units that can support a person in grief, and how have you collaborated with them? |

**Examples of Follow-up Questions**

- Could you tell me more about that?
- How do you think about that?
- What does that mean to you?
- Could you give an example?
- How do you feel about this?
- If I understand you correctly...
